# 

**Published:** 1887-08

**Authors:** 


					﻿sarrrwgsy hafst laji
Vol. III	
AUGUST 1887
No. 1
DANIEL'S
MEDICAL
JOURNAL
E. F. Danbar M.D. adsre kahsy
Noertahen hagsr
daSWE FADSR hafgst jahsr
				

